# Cervical cancer in the Bamenda Regional Hospital, North West Region of Cameroon: a retrospective study

**DOI:** 10.11604/pamj.2019.32.90.18217

**Published:** 2019-02-26

**Authors:** Ngwayu Claude Nkfusai, Samuel Nambile Cumber, Takang Williams, Judith K Anchang-Kimbi, Brenda Mbouamba Yankam, Cho Sabastine Anye, Joyce Mahlako Tsoka-Gwegweni, Enow-Orock George Enow, Damian Nota Anong

**Affiliations:** 1 Department of Microbiology and Parasitology, Faculty of Science, University of Buea, Buea, Cameroon; 2 Cameroon Baptist Convention Health Services (CBCHS), Yaoundé, Cameroon; 3 Faculty of Health Sciences, University of the Free State, Bloemfontein, South Africa; 4 Section for Epidemiology and Social Medicine, Department of Public Health, Institute of Medicine (EPSO), The Sahlgrenska Academy at University of Gothenburg, Gothenburg, Sweden; 5 School of Health Systems and Public Health Faculty of Health Sciences, University of Pretoria Private Bag X323, Gezina, Pretoria, 0001, Pretoria, South Africa; 6 Faculty of Health Sciences, University of Bamenda, Bamenda, Cameroon; 7 Department of Zoology and Animal Physiology, Faculty of Science, University of Buea, Buea, Cameroon; 8 Department of Statistics, Faculty of Physical Science, University of Nigeria, Nsukka, Nigeria; 9 School of Nursing & Public Health, College of Health Sciences, University of KwaZulu-Natal Durban, South Africa; 10 Faculty of Health Sciences, University of Buea, Buea, Cameroon; 11 Department of Biological Sciences, Faculty of Science, University of Bamenda, Bamenda, Cameroon

**Keywords:** Cervical cancer, retrospective study, Regional Hospital Bamenda

## Abstract

**Introduction:**

Cervical cancer is ranked the 7^th^ most common cancer in the world. Cancer of the cervix is the second most commonly diagnosed cancer after breast cancer and the third leading cause of cancer deaths among females in less developed countries. Incidence rates are highest in countries with low income. Nearly 90% of cervical cancer deaths occur in developing parts of the world. The study researchers therefore, carried out a retrospective study to determine the proportion of cervical cancer among other types of cancer in the cancer registry of the Bamenda Regional Hospital.

**Methods:**

The objective of this study was to determine the proportion of cervical cancer among other types of cancers in the cancer registry of the Bamenda Regional Hospital, North West Region of Cameroon from past records. We reviewed all records from the registry of patients who attended the Bamenda Regional Hospital to screen and/or be operated upon for cervical cancer and other types of cancer. Socio-demographic and clinical characteristics of cases were captured using a data collection sheet: age, type of cancer, stage of cancer, type of surgery carried out and date of surgery. Data were entered and analysed in Statistical Package for Social Sciences (SPSS) version 25 software.

**Results:**

59 cancer cases were received in the center between 2012 and 2017. Of these, 31 (52%) had cervical cancer. Most patients who screened positive for cancer of the cervix were of the 50-54 age groups. Most of these patients (47.5%), were received at late stages (stages 3 and 4).

**Conclusion:**

Over half (52%) of the patients receiving cancer care in this center have cervical cancer and generally turn up late for management.

## Introduction

Cervical cancer is ranked the 7^th^ most common cancer in the world. Cervical cancer is the second most commonly diagnosed cancer after breast cancer and the third leading cause of cancer deaths among females in less developed countries. Incidence rates are highest in countries with low income. Nearly 90% of cervical cancer deaths occurred in developing parts of the world. The cervix is divided into two halves; the endo-cervix, which is made up of columnar epithelium and the exo-cervix made up of squamous epithelium. The two halves merge at the squamo-columnar junction (SCJ), an important zone for malignant transformation. The SCJ varies in position throughout the reproductive life of a woman with an outward trend into the vagina. Cervical dysplasia, precancerous lesions in the cervical transformation zone can lead to cervical cancer [1]. Cervical cancer is preventable and curable if early diagnosed. The causative agent of the disease is the Human Papilloma Virus (HPV) which is transmitted through sexual intercourse causing cervical cancer through a slow growth over a period of 10-20 years [1]. There are over 150 different types of HPV; more than 40 can infect the cervix and are sexually transmitted, causing 99.4% of cervical cancer cases and 100% of genital warts cases. Out of the 150 types, about a dozen are carcinogenic. HPV-16 accounts for 50% to 55% of all cases of invasive cervical cancer worldwide while HPV-18 accounts for an additional 10% to 15%. Highly Active Antiretroviral Therapy (HAART) and evolution of cervical disease causes a high prevalence of cervical cancer in Cameroon [2]. Strains 16 and 18 are known to be responsible for up to 70% of cervical cancer worldwide [3]. It has been proven that, at least 50% of women who are sexually active have suffered an infection with at least one strain of HPV [4]. The global incidence of cervical cancer is greater than 530 000 annually, with death approaching 275 000 per year [5]. The prevalence of cervical cancer worldwide is estimated by [6] to be 12%. One of the most important reasons for the incidence of cervical cancer in developing countries is the lack of early detection of pre-cancerous lesions and treatment of the lesions before they progress [7].

Among the newly diagnosed cases, 86% are reported in poor countries. Also, 88% of deaths resulting from cervical cancer are in the low-income countries [8]. In Africa, the incidence is 80 000 per annum, with an annual mortality of 75%; most of the cases are seen in sub-Saharan Africa [8]. It was shown that cervical cancer prevalence was up to 13.8%; this is based on a study carried out in the capital city Yaounde [9]. More than 6 million Cameroonian females who are aged 15 and above are at risk of developing cervical cancer, and there are 1993 new cases of cervical cancer yearly, of which 1120 die of the disease annually [3]. In poor countries, awareness as well as uptake of cervical cancer screening services has remained poor over the years. Several studies done in communities and among women in sub-Saharan Africa revealed that knowledge were generally poor [10]. Risk factors of cervical cancers have also been highly demonstrated among Cameroonian women, especially the rural women [11]. Mogtomo and colleagues [12] have demonstrated a high incidence of sexually transmissible infections, multiple sexual partners, low use of condoms and other risk factors of cervical cancer among students in the University of Douala [13]. This therefore, calls for relevant measures to reduce this trend of progression. With current and appropriate measures put in place to prevent cervical cancer, success of screening will largely depend on the awareness and beliefs among women. The knowledge level of the general population is very important in determining the right strategy in planning an effective intervention against cervical cancer. WHO [2] reported that cervical cancer screening coupled with immediate management leads to early detection of precancerous and cancerous cervical lesions, thus preventing serious morbidity and mortality due to the cervical cancer disease' In a study carried out by [14-17], they demonstrated that the level of awareness of HPV infection and prevention of cervical cancer is moderately low in Cameroon. Gardasil for HPV 6 and 11, and Cervarix for HPV 16 and 18 are available in the market for cervical cancer [17].

## Methods

**Study design, setting and population:** The study was conducted in the Bamenda Regional Hospital, North West Region of the Republic of Cameroon. Bamenda is a city in North Western Cameroon and capital of the North West Region. The city has a population of about 500,000 people. Bamenda is situated at 5.95° North latitude, 10.16° East longitude and 1472 meters elevation above the sea level. In the cancer department where registration, screening and surgery for cancer are done is one of the 18 units of the Bamenda Regional Hospital. These units include, the Directorate and its Secretariat, Laboratory department, Day Care (which capture all HIV/AIDS, testing and treatment of cases), Diabetic/ Hypertensive unit, Ear, Nose and Throat Department, Dental Department, Out Patient Department, Social Service, Ostectric/Gynachologic, Theatre, General/Infant Consultation Department, Equipment Department, Sanitary Department, Mortuary, Palliative Care and the Obstetric/Gynecologic Department.

### Selection criteria

**Inclusion criterion:** All women managed for cancer from 2012 to 2017 in the centre were included in the final analysis.

**Exclusion criterion:** All negative results.

**Sample size and sampling:** All patients' records were checked.

**Data collection:** All patient's files were thoroughly checked and data on age, sex, type of cancer, type of surgery carried out and the year of surgery were captured into an excel template.

**Data analyses:** Data was entered into Excel, verified for completeness. Incomplete entries were deleted and cleaned before analyses. Descriptive data analysis in the form of tables, pie charts, and bar or column charts were used.

**Ethical considerations:** Ethical clearance was obtained from the Institutional Ethical Review Board of the Faculty of Health Sciences, University of Buea. Authorization to use the cancer registry was gotten from the Directorate of the Regional Hospital Bamenda who are the custodians of the cancer registry. Confidentiality and integrity of the data were maintained by restricting access of the information and primary data to the Principal Investigator.

## Results

**Figure 1:**
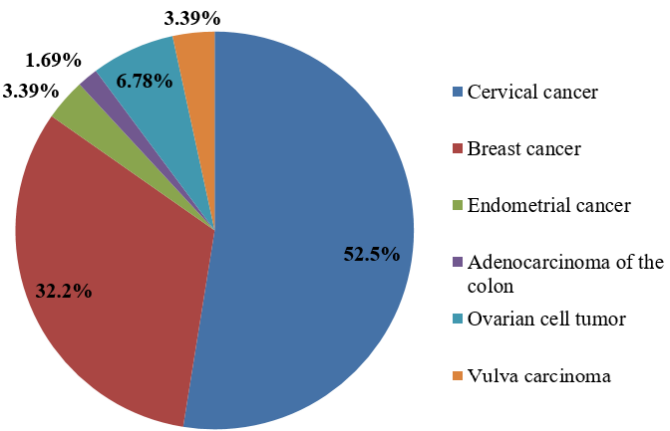
Proportions of different cancer types in patients consulting at the cancer registry of Bamenda Regional Hospital

**Availability of cancer data:** 59 cancer cases were received during the study period (2012 to 2017). Figure 1 shows the proportions of the different cancer types in patients who consulted at the cancer centre of the Bamenda Regional Hospital.

**Cancer types and types of surgery:** 6 different types of cancers (cervical cancer, breast cancer, endometrial cancer, adenocarcinoma of the colon, ovarian cell tumor and vulva carcinoma) were diagnosed within this period. Abdominal hysterectomy was the most common type of surgery done for all the cancers. The surgeries were carried out within the study period (Table 1, Figure 1).

**Table 1:** Cancer types, type of surgery and the year of surgery

S/N	Age	Type of cancer	Stage	Type of Surgery	Date of Surgery
1	31	Severe cervical displasia	CIN III(Cervical intraepithelial neoplasis	Total abdominal hysterectomy	26/11/14
2	44	Precancerous lesions of the cervix plus urinary incontinence	CIN III	TAH plus Burgs culpud suspension	18/11/16
3	42	Cervical neoplasia	Stage 1B2	Total abdominal hysterectomy	09/12/14
4	38	Severe cervical dysplasia	CIN III	Total abdominal hysterectomy	13/12/14
5	42	Endometrial carcinoma plus uterine fibroid	Stage 1	Total abdominal hysterectomy	06/01/15
6	69	Cervical cancer	Invasive cervical cancer stage 1B	Wertheims hysterectomy	06/02/15
7	43	Non keratinized moderaretly differentiated squamous carcinoma	Stage 1B	Wertheims hysterectomy	20/12/16
8		Cancer of cervix	Stage 2B	EUA	20/12/16
9	53	Precancerous lesions of the cervix	CIN III	laparatomy	27/03/15
10	20	Ovarian tumor	Stage 3	Exploratory laparatomy	17/01/12
11	40	Cervical cancer	Cervical dysplasia CIN3	HYSTERECTOMY	21/02/12
12	40	Breast cancer	T2NoMo	Radical left mastectomy	21/04/16
13	44	Breast cancer	T2N1N0	Radical left mastectomy	24/01/13
14	69	Cervical cancer	Carcinoma insitu	Total abdominal hystrectomy	24/10/12
15	50	Breast cancer	T2NoMo	mastectomy	13/03/13
16	48	Cervical cancer	Stage 1A	Total hysterectomy	02/06/16
17	37	Breast cancer	Intramedullary carcinoma of left breast	Left total mastectomy and axillary clearance	04/06/13
18	37	Bilateral breast cancer	T3N2M2	Bilateral mastectomy and lymph	10/08/16
19	55	Cervical cancer	Stage 1A	Total abdominal hysterectomy	15/07/13
20	60	Breast neoplasia	T2NoMo	Left total mastectomy	24/09/13
21	36	Breast cancer	T2NoMo	Mastectomy plus axillary clearance	14/11/13
22	22	Lobular carcinoma	T4N1MO	Radical mastectomy	31/10/16
23	51	Pre cervical cancer	CIN III	Total hysterectomy	09/11/16
24	44	Vagina, vulva plus genital warts	Stage2B	vulvectomy	########
25	54	Cervical cancer	Stage 2B	Total abdominal hysterectomy	########
26	34	Cervical cancer	2A	Weitheim hysterectomy	########
27	50	Cervical cancer	1B	Weitheim hysterectomy	########
28	54	Cervical cancer squamous cell carcinoma	2B	Wertheim hysterectomy	########
29	34	Cervical cancer	4	Radio/chemo	########
30	58	Adenocarcinoma colon		chemo	########
31	50	Cervical cancer	4	Radio/chemo	########
32	33	Cervical Cancer	4	quadrectomy	########
33	61	Cancer of cervix	4	Radio/chemo	########
34	52	Cervical cancer	Stage 4	Radio/ chemo	Rip
35	47	Cancer of the right breast	Stage 1	Quandrantectomy adenomectomy chemo, radiotherapy	########
36	46	CA of right breast invasive ducts carcinoma	1B	Mastectomy plus lymphadencetomy	########
37	43	Cervical cancer	1B	TAH	########
38	62	Cervical cancer	1B	TAH	########
39	23	Cancer of the right breast plus pregnacy	T3NoMo	Lost to follow-up	########
40	44	Precancerous lesions of the cervix	CIN2/CIN3	Total hysterectomy	05/01/17
41	42	Suspected left ovarian tumor	Stage 2	Total abdominal hysterectomy +BSO	20/01/17
42	54	Epidermoid carcinoma	Stage 1B	Total hysterectomy, wertheims hysterectomy	31/01/16
43	28	Left breast cancer	T2NoMo	Total left mastectomy	08/02/17
44	39	Cervical lesion suspected malignant		Total hysterectomy	27/01/17
45	46	Cervical cancer	CIN3	Weitheim hysterectomy	31/07/16
46	80	Endometrial hypeplasia in menopose	Stage I	Total abdominall hysterectomy	15/07/15
47	56	Precancerous cervix	CIN III	Total hysterectomy	26/06/15
48	68	Moderate cervical dysplasia	CIN II	Total hysterectomy	28/05/15
49	63	Granulosa cell tumor	Stage 2	Dibulking surgery	########
50	52	Squamous cell carcinoma	Stage I A	Total abdominal Hysterectomy	########
51	48	Breast cancer	T2N0M0	mastectomy	########
52	70	Granulosa cells tumors	Stage 2	Debulking surgery	########
53	50	Ductal carcinoma	T3N0M1	TAH	########
54	62	Aderno carcinpoma	Stage 1B	TAH +Radio therapy	########
55	47	Breast cancer	T2N	Mastectomy and radiotherapy	########
56	36	Ductal carcinoma	T4N1M1	Ductal chemotherapy	########
57	38	Breast lump	T2N1Mx	mastectomy	########
58	70	Breast cancer	T3N1Mx	mastectomy	########
59	40	Ductal carcinoma	T3N1Mx	mastectomy	########

**Figure 2:**
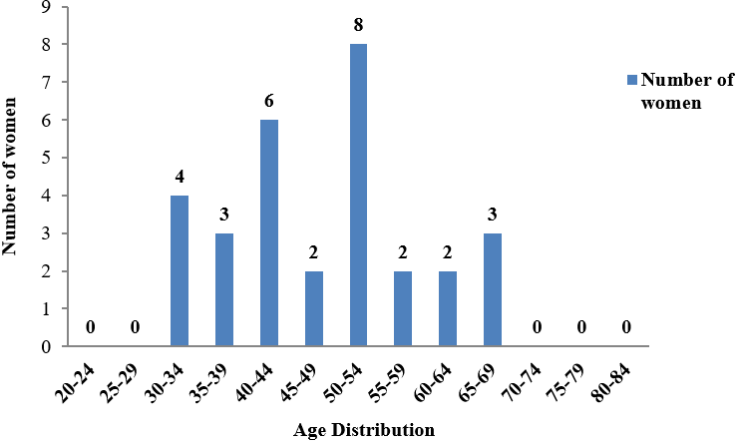
Cervical cancer distribution in patients of various ages consulting at the cancer registry of Bamenda Regional hospital

**Cervical cancer types and stages:** Cancer of cervix, cervical lesion, precancerous cervix, squamous cell carcinoma and cervical dysplasia were the types of cervical cancer, we found cancer of the cervix occuring the highest with 16 out of the 31 cases. Stage III was the most frequent stage of cervical cancer diagnosed (Table 2). Cervical cancer ranked first in all cases reported during this period with 31(52%) cases (Figure 2).

**Table 2:** Cervical cancer types and stages

S/N	Age	Type of Cancer	Stage
1	31	Severe cervical displasia	CIN III(Cervical intraepithelial neoplasis
2	44	Precancerous lesions of the cervix plus urinary incontinence	CIN III
3	42	Cervical neoplasia	Stage 1B2
4	38	Severe cervical dysplasia	CIN III
5	69	Cervical cancer	Invasive cervical cancer stage 1B
6	43	Non keratinized moderately differentiated squamous carcinoma	Stage 1B
7	45	Cancer of cervix	Stage 2B
8	53	Precancerous lesions of the cervix	CIN III
9	69	Cervical cancer	Carcinoma insitu
10	40	Cervical cancer	Cervical dysplasia CIN3
11	48	Cervical cancer	Stage 1A
12	55	Cervical cancer	Stage 1A
13	51	Pre-cervical cancer	CIN III
14	34	Cervical cancer	2A
15	50	Cervical cancer	1B
16	54	Cervical cancer squamous cell carcinoma	2B
17	34	Cervical cancer	4
18	50	Cervical cancer	4
19	33	Cervical Cancer	4
20	61	Cancer of cervix	4
21	52	Cervical cancer	Stage 4
22	43	Cervical cancer	1B
23	62	Cervical cancer	1B
24	39	Cervical lesion suspected malignant	CNIII
25	46	Cervical cancer	CIN3
26	56	Precancerous cervix	CIN III
27	68	Moderate cervical dysplasia	CIN II
28	52	Squamous cell carcinoma	Stage I A
29	36	Ductal carcinoma	T4N1M1
30	44	Precancerous lesions of the cervix plus urinary incontinence	CIN III
31	52	Squamous cell carcinoma	Stage I A

**Age distribution of cervical cancer:** The youngest patient was 31 years old while the oldest patient was 69 years. Most cases were between the ages of 50-54 years. The minimum age group of cervical cancer was 30-34years while the maximum was 65-69 years with the highest number of cervical cancer cases found in the 50-54 years age group (Figure 2).

## Discussion

Cancer of the cervix was the predominant type of cancer (52.2%) in the Bamenda Regional Hospital. This was far higher than 13.8% reported by [9] 2012 in Yaounde-Cameroon, and 29.9% reported by [17] 2013 in Yaounde-Cameroon. Their study results were derived from pathology results while ours was derived from surgery. It was also, different from a study conducted in 6 regions in Cameroon to determine the prevalence of cervical premalignant lesions from where; the national prevalence was 3.9% [15]. This difference could be due to the fact that, Tebeu and colleagues [15] had a larger sample size than our study and their study was representative. One of the most important reasons for the incidence of cervical cancer in developing countries is the lack of early detection of pre-cancerous lesions and treatment of the lesions before they progress [7].

Atashili and colleagues [16] also, reported a prevalence of 11.5% of SIL due to cervical disease in women receiving HAART in Cameroon. This was far lower than the 52% obtained in our study, it could however be different because our study was not limited to HIV/AIDS patients receiving HAART. Torre and colleagues [7] reported that cervical cancer is the second most commonly diagnosed cancer after breast cancer and the third leading cause of cancer deaths among females in less developed countries. From our study, cervical cancer ranked first, followed by breast cancer. This difference could be due to the fact that our study concentrated on just one region while that of Torre and colleagues [7] was carried out in several other regions. The highest age group with cervical cancer was 50-54; this is similar to the age at high risk of many types of cancer. Completeness of registration of cancer cases in this population was estimated at about 50%.

**Some limitations in our study includes:** Limited demographic information available for the women who were screened positive for cervical cancer; the fact that the researchers did not screen the women for cervical cancer but rather collected data from hospital records to determine the prevalence of cervical cancer is the reason for limited demographic information. Despite this shortcoming, this study provides relevant information in the context of very limited epidemiological data on the prevalence of cervical cancer among other types of cancers in the cancer registry of the Bamenda Regional Hospital, North West Region of Cameroon.

## Conclusion

The proportion of cervical cancer (52 %), among other types of cancers found in the cancer registry of the Bamenda Regional Hospital is high. The most affected group with cervical cancer was 50-54 year old group; which is similar to the age with a high risk to develop most cancers.

### What is known about this topic

- Cameroon in the year 2000, it revealed a prevalence of 7.9%, and the age of first intercourse as the major risk factor (the prevalence of cervical cancer in Cameroon in 2000 stood at 7.9%, which main risk factor being age at first sexual intercourse);
- Cervical cancer is the second most commonly diagnosed cancer after breast cancer 80 000 per annum, with an annual mortality of 75%; with most of the cases are seen in sub-Saharan Africa;
- More than 6 million Cameroonian females who are aged 15 and above are at risk of developing cervical cancer, and there are 1993 new cases of cervical cancer yearly, of which 1120 die of the disease annually.

### What this study adds

- Over half (52%) of cancers managed in the Bamenda Regional Hospital are cervical cancers;
- Most cancer patients seen are above 50 years of age;
- Most cancer cases are referred late in the disease course.

## Competing interests

The authors declare no competing interests.
